# Personal and family application of cancer prevention practices among physicians

**DOI:** 10.1038/s41598-026-66073-4

**Published:** 2026-08-18

**Authors:** Sarah S. Nasr, Nora Atef, Dalia Abdelfatah

**Affiliations:** Present Address: Cancer Epidemiology and Biostatistics Department, National Cancer Institute, Cairo University, Cairo, Egypt

**Keywords:** Cancer prevention, Physicians, Screening practices, Health Belief Model, Public health, Cancer, Health care, Oncology

## Abstract

Healthcare providers play a pivotal role in cancer prevention through screening advocacy, lifestyle counseling, and risk communication. However, limited evidence exists on whether physicians apply these preventive practices to themselves and their families. This study assessed personal and family-oriented cancer prevention behaviors among physicians and explored behavioral determinants using the Health Belief Model (HBM), with particular attention to differences between oncology-related and non-oncology specialties. A cross-sectional study was conducted among 353 physicians working at the National Cancer Institute, Cairo University, and affiliated hospitals across Cairo and Lower Egypt. Data were collected using a self-administered questionnaire assessing lifestyle risk factors, vaccination and screening practices, family-oriented prevention behaviors, perceived barriers, and HBM constructs. Comparisons between oncology-related and non-oncology specialties were performed using appropriate statistical tests. Although awareness of cancer warning signs was high (84.4%), participation in personal cancer screening was consistently low. Hepatitis B vaccination coverage was high (82.4%), whereas HPV vaccination uptake was limited (5.7%). Overweight, obesity, and physical inactivity were prevalent. Physicians working in oncology-related specialties demonstrated greater awareness of cancer warning signs and stronger engagement in family-oriented prevention activities but did not exhibit higher personal screening uptake. HBM construct scores were generally moderate. Younger physicians reported higher self-efficacy compared to older physicians (*p =* 0.005), while oncology-related practice was associated with higher perceived susceptibility and cues to action compared to non-oncology related specialties (*p* = 0.019&0.036 respectively). Perceived barriers were modest and did not significantly differ by specialty. Despite high professional awareness, physicians demonstrated limited engagement in personal cancer screening and preventive practices. Oncology-related practice enhanced preventive advocacy toward family members but did not translate into improved personal behaviors. These findings highlight a persistent knowledge–practice gap and underscore the need for institutional strategies that facilitate physician wellness, screening access, and preventive engagement.

**Supplementary Information:**

The online version contains supplementary material available at 10.1038/s41598-026-66073-4.

## Introduction

Cancer remains one of the leading causes of morbidity and mortality worldwide. In 2022, the World Health Organization estimated 19.3 million new cancer cases and nearly 10 million cancer-related deaths globally^1^. Early detection and prevention strategies including lifestyle changes, screening programs, and vaccination have been shown to substantially reduce the disease burden. For instance, modeling studies estimate that a 10% increase in adherence to recommended cancer screening guidelines could prevent thousands of deaths annually from breast, colorectal, cervical, and lung cancers^2^.

Healthcare providers (HCPs), as trusted health influencers, play a vital role in delivering preventive services and advocating for healthy behaviors. Their professional recommendations significantly shape patient choices. For example, trust in clinician advice has been associated with cancer screening adherence rates as high as 90% among eligible populations^3^. However, disparities persist: while 82.9% of U.S. primary care physicians perceive colonoscopy as “very effective,” only 77.9% routinely recommend it to patients; similar discrepancies are found for mammography, Pap smear, and PSA-based screening^4^.

Yet, less attention has been paid to whether healthcare providers themselves extend preventive cancer practices to their own families. Studies focusing on family history awareness reveal that around 39%–56% of individuals report discussing family cancer history with their providers^5^. Furthermore, clinicians’ personal behavior and attitudes may influence their professional practice. In one study of 900 HCPs, 91.4% affirmed the protective value of regular cancer screening, but only 3.5% had undergone colonoscopy themselves, and merely 15.9% of eligible women had received timely mammography^6^. This “knowing–doing gap,” whereby individuals recognize the importance of prevention yet fail to practice it suggests that professional awareness does not always translate into personal action.

The observed discrepancy between physicians’ professional knowledge and their personal engagement in cancer prevention suggests that knowledge alone is insufficient to promote sustained preventive behaviors. The Health Belief Model (HBM) provides a well-established theoretical framework for explaining this knowledge–practice gap by proposing that health behaviors are determined by perceived susceptibility, perceived severity, perceived benefits, perceived barriers, self-efficacy, and cues to action. Among physicians, these perceptions may influence whether extensive professional knowledge is translated into personal participation in cancer prevention and screening. Moreover, because physicians are trusted sources of health information and frequently influence health-related decisions within their households, the same HBM constructs are also likely to shape their promotion of cancer prevention among family members. Accordingly, the present study adopts the HBM as its conceptual framework to examine both physicians’ personal cancer prevention practices and their family-oriented preventive behaviors, providing a unified theoretical basis for understanding behavioral determinants across both domains^7^.

Communicating family cancer risk is essential not only for hereditary syndromes but also for broader prevention initiatives. Research shows that sharing cancer risk information with first-degree relatives is common, especially among female relatives (84–90%), yet less so for male relatives (53–62%)^8^. Effective intra-family communication increases awareness and promotes screening among at-risk relatives^9^. However, barriers such as logistical difficulties, lack of awareness, and emotional strain often hinder these discussions^10^.

Although physicians play a critical role in promoting cancer prevention and early detection among patients, relatively little is known about how they translate this knowledge into preventive behaviors within their own lives and families. Physicians represent a unique population because they possess advanced medical knowledge, routinely counsel patients on cancer prevention, and are widely regarded as trusted sources of health information. Consequently, they are expected not only to adopt recommended preventive practices themselves but also to influence the health behaviors of those closest to them. Within families, physicians often provide guidance on cancer risk factors, screening, vaccination, and healthy lifestyles, making the household an important extension of their preventive role. Examining both personal and family-oriented cancer prevention practices therefore provides a more comprehensive understanding of whether professional knowledge is translated into real-world preventive behaviors beyond the clinical setting. Despite this important role, evidence regarding physicians’ influence on cancer prevention within their families remains limited, highlighting an important gap in the literature that the present study seeks to address.

The primary aim of this study was to assess physicians’ engagement in personal and family-oriented cancer prevention practices. Secondary objectives were to: (1) examine behavioral determinants and perceived barriers influencing these practices using the Health Belief Model (HBM), and (2) compare cancer prevention behaviors and perceptions between physicians working in oncology-related specialties and those in non-oncology specialties. Through these objectives, the study seeks to provide insights into factors influencing physicians’ adoption and promotion of cancer prevention strategies.

### Study methodology

This multicenter cross-sectional study was conducted between November 2025 and March 2026 in Cairo and Lower Egypt. Participants were recruited from the National Cancer Institute, Cairo University, as well as governmental hospitals providing oncology and general medical services. Formal coordination was established with participating institutions to facilitate physician recruitment and data collection. The inclusion of multiple healthcare settings aimed to capture physicians from diverse specialties and practice environments, thereby improving the representativeness of the study population.

A convenience sampling technique was employed. Eligible physicians with at least one year of post-graduation clinical experience were invited to participate. This criterion was applied to ensure that participants had sufficient professional exposure to patient care and preventive health practices. Physicians from both oncology-related and non-oncology specialties were eligible for inclusion. The survey was distributed through institutional communication channels, professional networks, departmental meetings, and direct distribution of the questionnaire link via social media (WhatsApp, Instagram, Facebook, and Twitter). Participation was voluntary, and informed consent was obtained from all participants prior to survey completion. Non-medical staff and medical students were excluded from the study. The survey followed the Checklist for Reporting Results of Internet E-Surveys (CHERRIES) and the STROBE Statement guidelines for reporting observational studies^10,11,12^.

### Sample size

The required sample size was calculated using Epi Info™ (7.2.6.0) for estimating a single population proportion. The calculation assumed a 95% confidence level, a margin of error of 5%, and an expected prevalence of cancer screening practice among healthcare providers was 75%, based on the findings of previous cross-sectional study^6^, which investigated a comparable physician population. The minimum required sample size was 350 participants. Ultimately, 353 physicians completed the survey, exceeding the calculated sample size.

Despite adopting a convenience sampling strategy, Recruitment from multiple institutions and the application of standardized eligibility criteria have reduced selection bias. together with the excellent response rate that increases confidence in the representativeness of the study sample and minimizes the potential impact of non-response bias.

### Theoretical framework

The Health Belief Model (HBM) served as the theoretical framework underpinning this study. The HBM informed the study design by guiding the selection of behavioral constructs, the development of the questionnaire, and the evaluation of physicians’ cancer prevention practices. The questionnaire was designed to assess the six HBM constructs—perceived susceptibility, perceived severity, perceived benefits, perceived barriers, self-efficacy, and cues to action—in relation to both physicians’ personal preventive behaviors and their family-oriented cancer prevention practices. The HBM also provided the conceptual basis for interpreting the observed relationships between health beliefs and preventive behaviors.

### Data collection tool

#### Questionnaire development and validation

The study questionnaire was developed based on an extensive review of the literature and the Health Belief Model (HBM) to assess physicians’ personal and family-oriented cancer prevention practices and the behavioral determinants underlying these practices. The questionnaire underwent content validation by a panel of experts in epidemiology, oncology, and public health, who evaluated the relevance, clarity, comprehensiveness, and appropriateness of the items. Their recommendations were incorporated into the final version of the questionnaire^13–17^. The HBM component comprised six constructs: perceived susceptibility (2 items), perceived severity (2 items), perceived benefits (2 items), perceived barriers (5 items), self-efficacy (2 items), and cues to action (2 items). Responses were measured using a five-point Likert scale ranging from strongly disagree to strongly agree. The questionnaire was modified to reflect the objectives of the current study and to address both personal and family cancer prevention practices among physicians.

Subsequently, a pilot study was conducted among physicians who were not included in the final study sample to assess the clarity, feasibility, comprehensibility, and administration time of the questionnaire. Minor modifications were made based on participants’ feedback to improve wording and clarity. The reliability of the questionnaire was evaluated by assessing the internal consistency of the HBM constructs using Cronbach’s alpha coefficients, which demonstrated acceptable reliability for all measured domains.

The questionnaire consisted of three sections. The first section collected participants’ sociodemographic and professional characteristics. The second section assessed physicians’ personal and family cancer history, lifestyle characteristics, and cancer prevention practices. The third section was developed based on the Health Belief Model (HBM) and evaluated the six HBM constructs—perceived susceptibility, perceived severity, perceived benefits, perceived barriers, self-efficacy, and cues to action—in relation to physicians’ personal and family-oriented cancer prevention behaviors.

### Validity and reliability

#### Content validity

The tool was developed after evaluating relevant literature and submitting the questionnaire to a panel of public health and oncology specialists. The tool is provided in (Annex 1), with scale ranging from 1 (Strongly disagree) to 5 (Strongly agree). Each domain score was calculated by summing the item scores and dividing by the number of items within the domain (to have maximum score of 5 in each domain). The instrument was piloted on a small sample (*n* = 40) to ensure clarity and linguistic appropriateness, as well as to estimate completion time. Data from the pilot study were utilized to assess the internal consistency of the multi-item constructs.

#### Internal consistency reliability

Cronbach’s alpha coefficient was used to determine the internal consistency of the model items. It ranges between 0 and 1, with higher values indicating more internal consistency. Cronbach’s alpha cutoff values were based on the guidelines published by George and Mallery (2003)^18^: ≥ 0.9 is considered “excellent”, 0.8–0.9 is “good”, 0.7 to 0.8 is “acceptable”, 0.6 to 0.7 is “questionable”, 0.5 to 0.6 is “poor”, and below 0.5 is “unacceptable”. However, for exploratory research, a Cronbach’s alpha value of 0.5 or above is acceptable. Nunnally (1967)^19^ proposed that in early studies, reliability coefficients of 0.5 to 0.6 would suffice. In the context of this exploratory investigation, Cronbach’s alpha values of 0.5 or higher were deemed appropriate. In our study, Cronbach’s alpha for all items is within acceptable levels (0.60–0.88). “Internal consistency reliability was assessed separately for each Health Belief Model construct using Cronbach’s alpha coefficient. Reliability estimates were calculated for perceived susceptibility, perceived severity, perceived benefits, perceived barriers, self-efficacy, and cues to action. Construct-specific Cronbach’s alpha values were reported to provide a detailed assessment of the internal consistency of each domain.” (Annex).

#### Statistical analysis

Data were analyzed using SPSS version 27. Numerical data were described as mean and standard deviation or median and range, as appropriate. Qualitative data were expressed as frequency and percentage. Data were explored for normality using Kolmogorov-Smirnov and Shapiro-Wilk test. The Cronbach’s α coefficient was used to assess the scale’s internal consistency. Comparisons between oncology vs. non-oncology specialty were done using the Student’s t test for normally distributed numeric variables or Mann-Whitney test for non-normally distributed numeric variables. Comparisons between more than 2 groups were performed by Kruskal-Wallis for non-normally distributed variables. Chi-square (Fisher’s exact) test was used to examine the relation between qualitative variables as appropriate. All testes were two-tailed. A p-value < 0.05 was considered statistically significant.

#### Ethical considerations

The study protocol was reviewed and approved by the Institutional Review Board (IRB) of the National Cancer Institute, Cairo University (Approval No. EB2509-504-123-203). Participation was voluntary, and all eligible physicians were informed about the study objectives, procedures, and their right to withdraw at any time without consequence. Written informed consent was obtained from all participants prior to enrollment. To ensure confidentiality, no personally identifiable information was collected, and all responses were anonymized and stored securely. Data were used exclusively for research purposes and were accessible only to the study investigators.

## Results

A total of 353 physicians participated in the study. The mean age of participants was 38 years, and females constituted nearly two-thirds of the sample. Most participants were married, and the vast majority resided in urban areas. Physicians from a wide range of medical specialties participated, with approximately three-quarters holding a postgraduate qualification (MSc or MD), most participant were working in university hospitals. More than half of the respondents were working in oncology or oncology-related fields, with a median professional experience of 10 years, as shown in (Table 1).

**Table 1:** Participants characteristics (*n* = 353).

	Mean ± SD
**Age (Years)**	38 ± 11
**BMI (Kg/m2)**	28.5 ± 6.0
	**Median (Range)**
**Years of clinical experience**	10 (1–48)
	**n = 353 (%)**
**Gender**	
Female	232 (65.7)
Male	121 (34.3)
**Marital status**	
Single	86 (24.4)
Married	251 (71.1)
Widowed	9 (2.5)
Divorced	7 (2)
**Residence**	
Urban	330 (93.5)
Rural	23 (6.5)
**Highest level of education**	
BSC	50 (14.2)
Fellowship	37 (10.5)
Master degree	123 (34.8)
MD degree	143 (40.5)
**Specialty**	
Internal medicine	76 (21.7)
Academic	64 (18.2)
Medical oncology	54 (15.4)
Surgery	54 (15.4)
Surgical oncology	13 (3.7)
Radiology/Nuclear medicine	26 (7.4)
General practitioner	22 (6.3)
Epidemiology/Public health	20 (5.7)
Dentistry	5 (1.4)
Pediatrics	14 (4)
Pediatric oncology	3 (0.9)
**working in oncology or cancer related specialty**	
Yes	180 (51)
No	173 (49)
**Working in university hospital**	
Yes	233 (66)
No	120 (34)
**Do you have a first degree relative with a history of cancer**	
Yes	173 (49)
No	180 (51)
**Smoking**	
Current smoker	11 (3.1)
None smoker	333 (94.3)
X- smoker	9 (2.5)
**Do you consume alcohol**	
Yes	2 (0.6)
No	351 (99.4)
**How often do you exercise**	
Always (daily)	2 (0.6)
From time to time (1–2 times per week)	125 (35.4)
Never or rarely	186 (52.7)
Sometimes (3–4 times per week)	36 (10.2)
Usually (5–6 times per week)	4 (1.1)
**Fiber rich food consumption**	
Always (daily)	37 (10.5)
From time to time (1–2 times per week)	107 (30.3)
Never or rarely	6 (1.7)
Sometimes (3–4 times per week)	150 (42.5)
Usually (5–6 times per week)	53 (15)
**Processed meat consumption**	
Always (daily)	2 (0.6)
From time to time (1–2 times per week)	160 (45.3)
Never or rarely	111 (31.4)
Sometimes (3–4 times per week)	58 (16.4)
Usually (5–6 times per week)	22 (6.2)
**Hepatitis B vaccination**	
No	62 (17.6)
Yes	291 (82.4)
**HPV vaccination**	
No	333 (94.3)
Yes	20 (5.7)
**Awareness of early warning signs of cancer**	
Yes	298 (84.4)
No	55 (15.6)

SD: Standard deviation.

Participants were almost evenly divided with regard to having a first-degree family history of cancer (49.0% vs. 51.0%). The prevalence of current smoking was low. Alcohol consumption was uncommon, reported by only 0.6% of the study population. The mean body mass index was 28.5 ± 6.0 kg/m². Physical inactivity was frequently reported, with more than half of participants indicating that they never or rarely engaged in exercise. In contrast, only a small proportion reported regular daily or near-daily physical activity. Dietary patterns showed moderate intake of fiber-rich foods, typically consumed one to four times per week, while processed meat intake was relatively low, with nearly three-quarters of participants consuming it once weekly or less (Table 1).

With regard to preventive measures, most participants reported having received hepatitis B vaccination (82.4%). In contrast, uptake of HPV vaccination was very limited, with only 5.7% reporting vaccination. The participants had low exposure to certain behavioral risk factors, such as smoking and alcohol use, but high levels of physical inactivity, overweight, and suboptimal HPV vaccination coverage (Table 1).

Awareness of early warning signs of cancer was high, reported by 84.4% of participants. Despite this, engagement in cancer screening practices was consistently low across all screening modalities. Mammography was reported by only 9.3% of eligible participants, while Pap smear screening was particularly uncommon (2.8%). Similarly, colonoscopy and fecal occult blood testing were each reported by fewer than 4% of participants. Use of PSA testing (2.3%) and low-dose CT for lung cancer screening (1.4%) was also rare. Screening for skin cancer and oral cavity cancer was infrequent, reported by 4.0% and 2.5% of participants, respectively.

More than half of participants reported encouraging cancer screening among their parents or siblings, whereas encouragement of partner screening was less frequently reported (43.9%). Most participants indicated that they provided advice to family members regarding cancer risk factors and reported efforts to reduce family members’ exposure to harmful environmental factors. However, monitoring of family vaccination status was relatively uncommon (22.7%). Approximately 60% of participants stated that their family members were aware of early warning signs of cancer and had received education regarding cancer risk and its prevention. (Table 2).

**Table 2:** Familial cancer prevention behavior among study participants (*n* = 353).

Preventive behavior	No *n* (%)	Yes *n* (%)
Encourage partner screening (*n* = 297) *	142 (47.8)	155 (52.2)
Encourage parent/sibling screening (*n* = 337) **	139 (41.2)	198 (58.8)
Provided advice to family on cancer risk factors	65 (18.4)	288 (81.6)
Limit exposure to harmful environmental factors	63 (17.8)	290 (82.2)
Monitor family vaccination	273 (77.4)	80 (22.7)
Family members aware of early warning signs of cancer	143 (40.5)	210 (59.5)
Educated family about cancer risk and prevention	136 (38.5)	217 (61.5)

* 56 patients were not applicable, ** 16 patients were not applicable.

Participants expressed strong general beliefs regarding the seriousness of cancer and the importance of prevention. Most agreed that cancer can affect individuals regardless of profession and acknowledged its serious consequences for themselves and their families. A large majority also believed that adherence to cancer prevention guidelines and participation in screening could reduce cancer risk and improve outcomes. In contrast, perceived personal susceptibility to cancer was relatively low. Half of participants reported neutral views regarding their own lifetime cancer risk, and less than one-third agreed that they were personally at risk. Confidence in their ability to consistently follow cancer prevention guidelines was moderate, with a substantial proportion expressing neutral responses. Approximately 78.5% of participants agreed or strongly agreed that cancer screening facilitates early detection and improves outcomes. However, substantially lower levels of self-efficacy were reported, with only about one-third of physicians expressing confidence in their ability to consistently engage in recommended cancer prevention and screening practices. Nearly half of the participants agree that they can influence family members’ health-related behaviors positively. (Table 3).

**Table 3:** Perception, barriers, and cues to action for cancer prevention practicing among study participants (*n* = 353).

Item	Strongly agree	Agree	Neutral	Disagree	Strongly disagree
Perception n (%)					
Believe I am at risk of developing cancer in my lifetime	27 (7.6)	84 (23.8)	177 (50.1)	44 (12.5)	21 (5.9)
Cancer could affect anyone regardless of profession	107 (30.3)	151 (42.8)	47 (13.3)	16 (4.5)	32 (9.1)
Cancer has serious consequences for my life and family	128 (36.3)	119 (33.7)	47 (13.3)	20 (5.7)	39 (11.0)
If I were diagnosed with cancer, my career would be negatively affected	84 (23.8)	121 (34.3)	72 (20.4)	36 (10.2)	40 (11.3)
Following cancer prevention guidelines can significantly reduce my risk	77 (21.8)	167 (47.3)	64 (18.1)	18 (5.1)	27 (7.6)
Screening helps detect cancer early and improves outcomes	138 (39.1)	139 (39.4)	30 (8.5)	9 (2.5)	37 (10.5)
I feel confident in my ability to follow cancer prevention guidelines	21 (5.9)	101 (28.6)	149 (42.2)	57 (16.1)	25 (7.1)
I can influence my family’s health-related behaviours positively	24 (6.8)	138 (39.1)	127 (36.0)	43 (12.2)	21 (5.9)
**Barriers n (%)**					
I don’t have time to engage in screening or healthy practices	18 (5.1)	87 (24.6)	123 (34.8)	87 (24.6)	38 (10.8)
I am concerned about the cost of cancer screening	35 (9.9)	87 (24.6)	111 (31.4)	88 (24.9)	32 (9.1)
I avoid screening because I don’t want to know bad news	18 (5.1)	50 (14.2)	82 (23.2)	126 (35.7)	77 (21.8)
Cancer screening services are inaccessible to family	13 (3.7)	51 (14.4)	88 (24.9)	147 (41.6)	54 (15.3)
I don’t believe screening is necessary unless symptoms appear	7 (2.0)	24 (6.8)	51 (14.4)	159 (45.0)	112 (31.7)
**Cues to action n (%)**					
I am more likely to act if I receive reminders from health campaigns	36 (10.2)	139 (39.4)	112 (31.7)	40 (11.3)	26 (7.4)
Seeing patients with cancer makes me more aware of prevention needs	65 (18.4)	153 (43.3)	82 (23.2)	25 (7.1)	28 (7.9)

Perceived barriers to cancer screening and healthy practices were generally moderate. Time constraints and financial concerns were reported by approximately one-third of participants, although a similar proportion did not view these factors as major barriers. Fear of receiving bad news was less prominent, with most participants disagreeing that this prevented them from undergoing screening. Additionally, most respondents did not perceive cancer screening services as inaccessible to their families, with the majority rejecting the notion that screening is only necessary once symptoms develop (Table 3).

Regarding cues to action for cancer prevention practices among physicians, reminders from health campaigns were reported as a motivating factor by nearly half of participants, with 49.6% either agreeing or strongly agreeing that such reminders increased their likelihood of acting. However, almost one-third (31.7%) expressed a neutral stance, while 18.7% disagreed or strongly disagreed. In contrast, direct clinical exposure appeared to be a stronger cue; a majority of physicians agreed or strongly agreed that seeing patients with cancer increased their awareness of prevention needs. Neutral responses were reported by 23.2% of participants, whereas 15.0% disagreed or strongly disagreed (Table 3).

Overall, the findings based on the Health Belief Model demonstrated a distinct pattern across the measured constructs. Participants exhibited moderate levels of perceived susceptibility to cancer, indicating limited personal perception of risk. In contrast, perceived severity and perceived benefits of cancer prevention and screening were generally high, reflecting strong recognition of the seriousness of cancer and the value of preventive practices. Perceived barriers were moderate, with time constraints and competing professional responsibilities being the most commonly reported obstacles. Notably, self-efficacy scores were comparatively lower than other HBM domains.

Table 4 presents the distribution of Health Belief Model (HBM) construct scores for physicians according to sociodemographic and professional characteristics. Median scores for all HBM constructs generally ranged between 2 and 3 across different subgroups. With respect to age, physicians aged ≤ 35 years and those older than 35 years demonstrated comparable median scores across most HBM constructs. However, a statistically significant difference was observed for self -efficacy, with younger physicians (≤ 35 years) reporting higher median scores compared to their older counterparts (median 3 vs. 2, *p* = 0.005).

**Table 4:** Health belief model constructs in relation to demographic and occupational characteristics of study participants (*n* = 353).

	Perceived Susceptibility	*P* value	Perceived Severity	*P* value	Perceived Benefits	*P* value	Perceived Barriers	*P* value	Self-Efficacy	*P* value	Cues to Action	*P* value
	Median (Range)		Median (Range)		Median (Range)		Median (Range)		Median (Range)		Median (Range)	
Age group												
≤ 35 Year	3 (1–5)	0.559	2 (1–5)	0.442	2 (1–5)	0.103	3 (1–5)	0.377	3 (1–5)	0.005*	3 (1–5)	0.324
> 35 Year	3 (1–5)		2 (1–5)		2 (1–5)		3 (1–5)		2 (1–5)		2 (1–5)	
**Gender**												
Male	3 (1–5)	0.414	2 (1–5)	0.895	3 (1–5)	0.037*	3 (1–5)	0.324	3 (1–5)	0.589	3 (1–5)	0.201
Female	3 (1–5)		2 (1–5)		2 (1–5)		3 (1–5)		3 (1–5)		3 (1–5)	
**Marital status**												
Single	3 (1–5)	0.025*	2 (1–5)	0.016*	2 (1–5)	0.059	3 (2–5)	0.105	3 (1–5)	0.313	3 (1–5)	0.131
Married	3 (1–5)		2 (1–5)		2 (1–5)		3 (1–5)		3 (1–5)		3 (1–5)	
Widowed	3 (2–5)		3 (2–5)		2 (1–5)		3 (2–5)		2 (1–5)		3 (1–5)	
Divorced	2 (1–3)		1 (1–2)		2 (1–3)		2 (2–4)		3 (1–4)		2 (2–2)	
**Residence**												
Urban	3 (1–5)	0.878	2 (1–5)	0.866	2 (1–5)	0.811	3 (1–5)	0.210	3 (1–5)	0.307	3 (1–5)	0.913
Rural	3 (2–5)		3 (1–5)		2 (1–5)		3 (2–5)		3 (2–5)		2 (2–5)	
**Highest level of education**												
BSC	3 (2–5)	0.027*	2 (1–5)	0.246	2 (1–5)	0.133	3 (2–5)	0.663	3 (1–5)	0.231	3 (1–5)	0.154
Fellowship	2 (1–5)		2 (1–5)		2 (1–5)		3 (1–5)		3 (2–5)		2 (1–5)	
Master degree	3 (1–5)		2 (1–5)		2 (1–5)		3 (1–5)		3 (1–5)		3 (1–5)	
MD degree	3 (1–5)		2 (1–5)		2 (1–5)		3 (1–5)		3 (1–5)		3 (1–5)	
**Specialty**												
Academic	3 (1–5)	0.005*	3 (1–5)	0.218	2 (1–5)	0.105	3 (2–5)	0.808	3 (1–5)	0.221	2.5 (1–5)	0.406
Medical oncology	2 (1–5)		2 (1–5)		2 (1–5)		3 (1–5)		3 (1–5)		2 (1–5)	
Epidemiology/Public health	2 (1–4)		2 (1–5)		2 (1–5)		3.5 (2–5)		3 (1–5)		2 (1–5)	
General practitioner	3 (2–5)		3 (1–5)		2.5 (1–5)		3 (1–5)		3 (1–5)		3 (1–5)	
Internal medicine	3 (1–5)		2 (1–5)		2 (1–5)		3 (1–5)		3 (1–5)		3 (1–5)	
Pediatrics	3 (2–5)		2 (1–5)		2 (1–5)		3 (2–5)		2.5 (2–4)		3 (1–5)	
Pediatric oncology	2 (2–2)		1 (1–2)		2 (2–2)		3 (3–3)		4 (3–4)		2 (1–3)	
Radiology/Nuclear medicine	2.5 (2–5)		2 (1–4)		2 (1–4)		3 (2–4)		3 (2–5)		3 (1–4)	
Surgery	3 (1–5)		2 (1–5)		2 (1–5)		3 (1–5)		3 (1–5)		2 (1–5)	
Surgical oncology	3 (1–5)		2 (1–5)		2 (1–5)		4 (2–5)		3 (2–5)		2 (1–5)	
Dentistry	2 (2–4)		2 (2–4)		2 (2–5)		3 (2–4)		3 (2–4)		2 (2–4)	
**Years of experience**												
≤ 10 Years	3 (1–5)	0.593	2 (1–5)	0.123	2 (1–5)	0.407	3 (1–5)	0.315	3 (1–5)	0.055	3 (1–5)	0.645
> 10 Years	3 (1–5)		2 (1–5)		2 (1–5)		3 (1–5)		3 (1–5)		3 (1–5)	
**Working in university hospital**												
Yes	3 (1–5)	0.515	2 (1–5)	0.521	2 (1–5)	0.165	3 (1–5)	0.146	3 (1–5)	0.677	3 (1–5)	0.659
No	3 (1–5)		2 (1–5)		2 (1–5)		3 (1–5)		3 (1–5)		3 (1–5)	
**Working in oncology or cancer related specialty**												
Yes	3 (1–5)	0.019*	2 (1–5)	0.125	2 (1–5)	0.060	3 (1–5)	0.517	3 (1–5)	0.494	3 (1–5)	0.036*
No	2.5 (1–5)		2 (1–5)		2 (1–5)		3 (1–5)		3 (1–5)		2 (1–5)	

* Statistically significant at p-value < 0.05.

Regarding gender, no statistically significant differences were found between males and females for perceived susceptibility, perceived severity, perceived barriers, self-efficacy, or cues to action. Nevertheless, perceived benefits differed significantly by gender, with male physicians reporting higher median scores than females (median 3 vs. 2, *p* = 0.037).

Analysis by marital status revealed statistically significant differences in both perceived susceptibility (*p* = 0.025) and perceived severity (*p* = 0.016). Single, married and widowed physicians showed similar median scores, while divorced physicians reported the lowest medians. No significant differences were observed across marital status for perceived benefits, perceived barriers, self-efficacy, or cues to action. No statistically significant differences were detected between urban and rural residents across any of the HBM constructs.

When examined by highest level of education, a statistically significant difference was found only for perceived susceptibility (*p* = 0.027), with physicians holding a bachelor’s degree reporting higher median scores compared to those with fellowship qualifications. No significant associations were observed between educational level and the remaining HBM constructs.

Significant variation in perceived susceptibility was also observed across medical specialties (*p* = 0.005). Academic physicians and general practitioners reported higher median scores, whereas lower medians were observed among medical oncology, epidemiology/public health, and pediatric oncology specialties. No statistically significant differences were found across specialties for perceived severity, perceived benefits, perceived barriers, self-efficacy, or cues to action.

Regarding years of experience, no statistically significant differences were identified between physicians with ≤ 10 years and those with > 10 years of experience across any HBM construct, although self-efficacy approached statistical significance (*p* = 0.055). Similarly, working in a university hospital was not significantly associated with any of the HBM construct scores.

Physicians working in oncology or cancer-related specialties demonstrated significantly higher perceived susceptibility (*p* = 0.019) and cues to action (*p* = 0.036) compared to those working in non-oncology specialties. No statistically significant differences were observed between the two groups for perceived severity, perceived benefits, perceived barriers, or self-efficacy (Table 4).

When comparing physicians working in oncology-related specialties with those in other fields, no statistically significant differences were observed for most lifestyle factors, including family history of cancer, smoking status, alcohol consumption, BMI, dietary fiber intake, processed meat consumption, and HPV vaccination status (*p* > 0.05). Physicians working in oncology-related specialties were significantly more likely to be employed at university hospitals and to hold postgraduate qualifications (Fellowship, MSc, or MD degrees). Also, a significant difference was noted in physical activity patterns, with oncology-related physicians reporting more frequent regular exercise (*p* = 0.013). Hepatitis B vaccination coverage was also significantly higher among physicians working in oncology-related specialties (*p* = 0.024). Overall, specialty type was not associated with most behavioral risk factors but was related to selected preventive practices (Table 5).

**Table 5:** Occupational characteristics, cancer risk profile, lifestyle, vaccination status, and family cancer prevention practices among physicians working in oncology-related versus non-oncology-related specialties.

Variable		Working in oncology or related specialty		
		No (*n* = 173) n (%)	Yes (*n* = 180) n (%)	*P* value
Working in University hospitals	No	72 (60.0)	48 (40.0)	0.003*
	Yes	101 (43.3)	132 (56.7)	
Highest level of education	BSC	32 (64.0)	18 (36.0)	0.035*
	Fellowship	12 (32.4)	25 (67.6)	
	Master degree	60 (48.8)	63 (51.2)	
	MD degree	69 (48.3)	74 (51.7)	
First-degree relative with history of cancer	No	93 (51.7)	87 (48.3)	0.308
	Yes	80 (46.2)	93 (53.8)	
Smoking	Current	3 (27.3)	8 (72.7)	0.356^f^
	Non-smoker	166 (49.8)	167 (50.2)	
	Ex-smoker	4 (44.4)	5 (55.6)	
Alcohol	No	172 (49.0)	179 (51.0)	1.000^f^
	Yes	1 (50.0)	1 (50.0)	
BMI	Mean (SD)	28.2 (5.9)	28.8 (6.1)	0.337
Exercise rate	Always (daily)	0 (0.0)	2 (100.0)	0.0129^f^
	Usually (5–6 times per week)	0 (0.0)	4 (100.0)	
	Sometimes (3–4 times per week)	21 (58.3)	15 (41.7)	
	From time to time (1–2 times per week)	63 (50.4)	62 (49.6)	
	Never/rarely	89 (47.8)	97 (52.2)	
Fiber-rich food consumption	Always (daily)	24 (64.9)	13 (35.1)	0.069
	Usually (5–6 times per week)	23 (43.4)	30 (56.6)	
	Sometimes (3–4 times per week)	64 (42.7)	86 (57.3)	
	From time to time (1–2 times per week)	58 (54.2)	49 (45.8)	
	Never/rarely	4 (66.7)	2 (33.3)	
Processed meat consumption	Always (daily)	1 (50.0)	1 (50.0)	0.877
	Usually (5–6 times per week)	10 (45.5)	12 (54.5)	
	Sometimes (3–4 times per week)	32 (55.2)	26 (44.8)	
	From time to time (1–2 times per week)	78 (48.8)	82 (51.3)	
	Never/rarely	52 (46.8)	59 (53.2)	
HBV vaccination	No	32 (64.0)	18 (36.0)	0.024*
	Yes	136 (46.7)	155 (53.3)	
	Not applicable**	5 (41.7)	7 (58.3)	
HPV vaccination	No	142 (47.7)	156 (52.3)	0.285
	Yes	12 (60.0)	8 (40.0)	
	Not sure**	19 (54.3)	16 (45.7)	
Encourage partner screening	No	83 (58.5)	59 (41.5)	0.003*
	Yes	64 (41.3)	91 (58.7)	
	Not applicable**	26 (46.4)	30 (53.6)	
Encourage parent/sibling screening	No	79 (56.8)	60 (43.2)	0.015*
	Yes	86 (43.4)	112 (56.6)	
	Not applicable**	8 (50.0)	8 (50.0)	
Provided advice to family on cancer risk factors	No	37 (21.4)	28 (15.6)	0.158
	Yes	136 (78.6)	152 (84.4)	
Limit exposure to harmful environmental factors	No	25 (14.5)	38 (21.1)	0.102
	Yes	148 (85.5)	142 (78.9)	
Monitor family vaccination	No	133 (76.9)	140 (77.8)	0.840
	Yes	40 (23.1)	40 (22.2)	
Family members aware of early warning signs of cancer	No	84 (48.6)	59 (32.8)	0.003*
	Yes	89 (51.4)	121 (67.2)	
Educated family about cancer risk and prevention	No	77 (44.5)	59 (32.8)	0.024*
	Yes	96 (55.5)	121 (67.2)	

f: Fisher exact, * Statistically significant at p-value < 0.05, ** Not included in comparison.

However, no significant differences were found between the two groups with regard to personal uptake of cancer screening practices, including mammography, Pap smear, colorectal cancer screening, PSA testing, low-dose CT, skin examination, or oral cavity examination (all *p* > 0.05).

Physicians in oncology-related fields were more likely to encourage screening among partners and parents or siblings, report greater family awareness of early warning signs, and indicate that they had educated family members about cancer risk and prevention (*p* < 0.05 for all). They were also more likely to report efforts to limit family exposure to harmful environmental factors. In contrast, no significant differences were observed between groups in providing advice on cancer risk factors or monitoring family vaccination status (Table 5).

Perceptions related to cancer risk, severity, prevention, and screening did not differ significantly between physicians working in oncology-related specialties and those in non-oncology fields (all *p* > 0.05). Both groups showed comparable perceptions of personal cancer risk, recognition of cancer seriousness, beliefs in the effectiveness of prevention and screening, confidence in following preventive practices, and perceived influence on family health behaviors (Table 6).

Similarly, no statistically significant differences were observed between oncology-related and non-oncology physicians regarding perceived barriers to cancer screening, including lack of time, cost concerns, fear of bad news, or perceived inaccessibility of screening services (all *p* > 0.05). Although a higher proportion of non-oncology physicians tended to agree that screening is unnecessary in the absence of symptoms, this difference did not reach statistical significance (*p* = 0.053) (Table 6).

**Table 6:** Health belief model in relation to working in oncology specialty.

Item	Do you work in oncology or related specialty?						p-value
	No (n = 173)			Yes (n = 180)			
	Strongly agree–Agree	Neutral	Strongly disagree–Disagree	Strongly agree–Agree	NeutralYes	Strongly disagree–Disagree	
**Perception n (%)**							
Believe I am at risk of developing cancer in my lifetime	47 (42.3)	88 (49.7)	38 (58.5)	64 (57.7)	89 (50.3)	27 (41.5)	0.260
Cancer could affect anyone regardless of profession	118 (45.7)	25 (53.2)	30 (62.5)	140 (54.3)	22 (46.8)	18 (37.5)	0.270
Cancer has serious consequences for my life and family	112 (45.3)	28 (59.6)	33 (55.9)	135 (54.7)	19 (40.4)	26 (44.1)	0.194
If I were diagnosed with cancer, my career would be negatively affected	95 (46.3)	40 (55.6)	38 (50.0)	110 (53.7)	32 (44.4)	38 (50.0)	0.508
Following cancer prevention guidelines can significantly reduce my risk	113 (46.3)	34 (53.1)	26 (57.8)	131 (53.7)	30 (46.9)	19 (42.2)	0.476
Screening helps detect cancer early and improves outcomes	126 (45.5)	20 (66.7)	27 (58.7)	151 (54.5)	10 (33.3)	19 (41.3)	0.094
I feel confident in my ability to follow cancer prevention guidelines	62 (50.8)	71 (47.7)	40 (48.8)	60 (49.2)	78 (52.3)	42 (51.2)	0.871
I can influence my family’s health-related behaviors positively	75 (46.3)	64 (50.4)	34 (53.1)	87 (53.7)	63 (49.6)	30 (46.9)	0.835
**Barriers n (%)**							
I don’t have time to engage in screening or healthy practices	55 (52.4)	58 (47.2)	60 (48.0)	50 (47.6)	65 (52.8)	65 (52.0)	0.856
I am concerned about the cost of cancer screening	61 (50.0)	49 (44.1)	63 (52.5)	61 (50.0)	62 (55.9)	57 (47.5)	0.610
I avoid screening because I don’t want to know bad news	31 (45.6)	37 (45.1)	105 (51.7)	37 (54.4)	45 (54.9)	98 (48.3)	0.742
Cancer screening services are inaccessible to family	35 (54.7)	47 (53.4)	91 (45.3)	29 (45.3)	41 (46.6)	110 (54.7)	0.518
I don’t believe screening is necessary unless symptoms appear	18 (58.1)	24 (47.1)	131 (48.3)	13 (41.9)	27 (52.9)	140 (51.7)	0.053
**Cues to action** **n (%)**							
I am more likely to act if I receive reminders from health campaigns	83 (47.4)	51 (45.5)	39 (59.1)	92 (52.6)	61 (54.5)	27 (40.9)	0.183
Seeing patients with cancer makes me more aware of prevention needs	103 (47.2)	38 (46.3)	32 (60.4)	115 (52.8)	44 (53.7)	21 (39.6)	0.197

despite high awareness of cancer warning signs, strong recognition of disease severity, and high belief in the benefits of prevention, physicians demonstrated remarkably low personal engagement in cancer screening practices and low self-efficacy regarding self-care. While high workplace exposure among oncology specialists significantly increased perceived risk, cues to action, and health promotion toward family members, it did not translate into improved personal screening uptake compared to non-oncology peers. Overall, time constraints and low self-efficacy emerged as primary obstacles, revealing a critical gap between professional medical knowledge and personal health behaviors among physicians.

## Discussion

This study examined cancer-related risk factors, preventive behaviors, perceptions, and advocacy practices among physicians, with particular attention to differences between oncology-related and non-oncology specialties. Despite a high level of professional knowledge, the findings demonstrate a persistent gap between awareness of cancer prevention and engagement in personal preventive and screening practices.

### Lifestyle factors and behavioral risks

Physicians in this study exhibited a low prevalence of smoking and alcohol consumption, consistent with previous literature demonstrating healthier behavioral profiles among physicians compared to the general population^20,21^. Dietary patterns were also generally favorable, characterized by moderate intake of fiber-rich foods and low consumption of processed meats—aligning with current nutritional guidelines aimed at mitigating colorectal cancer risk^24^.

However, excess body weight (mean BMI: 28.5 ± 6 kg/m^2)^ and physical inactivity were highly prevalent, with over half of respondents reporting that they rarely or never engage in exercise. These findings are concerning, given the established physiological pathways linking high body fat and sedentary behavior to chronic inflammation and increased risk for multiple malignancies. Similar adverse lifestyle patterns have been widely reported among healthcare professionals and are frequently attributed to heavy occupational demands, extended work hours, shift work, and limited opportunities for recreational physical activity^22,23^. Interestingly, sub-analysis in our study revealed that physicians in oncology-related specialties engaged in regular physical activity significantly more often than non-oncology peers (*p* = 0.0129), suggesting that daily proximity to cancer care may reinforce personal commitment to modifiable lifestyle habits, even when overall time constraints remain an obstacle.

### Vaccination status and cancer screening practices

Hepatitis B vaccination coverage was high, particularly among physicians in oncology-related fields, likely reflecting institutional policies and occupational safety mandates^25^. In contrast, uptake of HPV vaccination was notably low (5.7%). This finding aligns with trends reported in low- and middle-income settings, where adult vaccination programs face structural barriers, including out-of-pocket costs and limited workplace delivery systems^26–28^.

Although most participants demonstrated high awareness of early warning signs of cancer, personal participation in cancer screening remained low. A plausible, documented explanation for this discrepancy is the concept of low personal risk perception, often discussed in literature as an “invincibility bias” or optimism bias among healthcare professionals^29^. Because physicians routinely act as care providers, they may unconsciously perceive themselves as less vulnerable to chronic diseases—a phenomenon reflected in our HBM findings where perceived personal susceptibility remained low to neutral despite high knowledge scores^29,30^. Furthermore, structural constraints within medical practice, such as rigid scheduling and fear of creating additional workload for colleagues, can create a culture where personal preventive health is deferred^31^. Qualitative literature among health professionals also suggests that concerns surrounding peer confidentiality and reluctance to adopt the “patient role” within one’s own workplace may serve as additional informal obstacles to formal screening protocols, though further qualitative research in this specific population is needed to confirm these dynamics^29,31^.

### Cancer-related perceptions and self-efficacy

Most physicians acknowledged the seriousness of cancer and the benefits of prevention and early detection. These findings are consistent with health behavior theories that emphasize perceived severity and perceived benefit as important determinants of preventive behavior^32^. However, perceived personal susceptibility to cancer was relatively low, with many participants expressing neutral responses reflecting optimism bias or professional desensitization due to frequent clinical exposure to cancer patients, as suggested in previous studies^33^.

### Health belief model constructs and determinants

The central tendency of Health Belief Model (HBM) construct scores toward moderate levels aligns with prior research among healthcare workers, reflecting a balanced, measured risk appraisal rather than polarized health beliefs^34^. Rather than uniform perceptions across the cohort, key demographic and professional factors significantly shaped individual HBM domains: Demographic and Life Experience Factors: The significantly higher self-efficacy observed among younger physicians (≤ 35 years) likely reflects recent exposure to modern, competency-based medical curricula that emphasize preventive decision-making and self-care^35^. Gender variations in perceived benefits, alongside elevated susceptibility and severity scores among widowed physicians, further demonstrate how gender socialization and significant personal life events shape individual risk perception and the perceived utility of preventive health^36,37^.Professional Exposure and Geographic Uniformity: The lack of urban–rural differences across HBM constructs suggests that standardized medical education and uniform clinical guidelines mitigate the geographic disparities in health literacy typically seen in the general population^38,39^. Notably, oncology specialists demonstrated significantly higher perceived susceptibility and cues to action, reinforcing existing literature that daily, immersive exposure to cancer patients serves as a potent cue that heightens personal risk perception^39^.Collectively, these findings affirm the utility of the HBM in capturing physicians’ health perceptions, highlighting that individual clinical exposure and career stage rather than geographic location are the primary drivers shaping preventive health beliefs.

### Impact of oncology-related practice

Physicians working in oncology-related specialties demonstrated significantly higher awareness of early warning signs of cancer compared with their non-oncology counterparts. However, this increased awareness was not associated with higher personal uptake of cancer screening. This finding is consistent with previous reports indicating that professional exposure to cancer does not necessarily lead to improved personal screening behaviors^40,41^.

In contrast, oncology-related practice was associated with greater engagement in family-oriented cancer prevention activities. Physicians in oncology-related specialties were more likely to encourage cancer screening among family members, promote awareness of early warning signs, and provide education on cancer risk and prevention^17^. These findings suggest that oncology exposure may strengthen preventive advocacy toward others, even when personal behaviors remain unchanged. This observation may reflect the impact of regular exposure to cancer patients and firsthand awareness of the consequences of delayed diagnosis. Such professional experiences may increase perceived susceptibility and perceived severity, thereby strengthening motivation to promote preventive behaviors. These findings highlight the potential value of targeted educational interventions aimed at improving cancer prevention practices among physicians in non-oncology specialties.

### Barriers, cues to action, and preventive engagement

Perceived barriers to cancer screening were generally moderate. Time constraints and cost were reported by some participants, whereas fear of diagnosis and access-related barriers were less frequently endorsed. These findings align with prior literature that has documented multifaceted barriers to cancer screening uptake across settings—including logistical challenges, financial constraints, and competing priorities—that can limit preventive practices even among healthcare providers and patients alike. Systematic reviews have consistently shown that practical barriers such as time pressure and perceived screening costs are commonly cited obstacles to screening participation and recommendation practices, reinforcing the need for structural and policy-level interventions to support preventive care delivery^42^.

Although physicians demonstrated high perceived severity of cancer and strong recognition of the benefits of screening and preventive practices, these factors alone were insufficient to promote consistent preventive behaviors. Participants generally reported moderate perceived susceptibility, suggesting that many physicians did not consider themselves personally at risk despite their professional knowledge of cancer. In addition, relatively low self-efficacy may have reduced their confidence in initiating and maintaining preventive behaviors, while practical barriers such as time constraints and competing professional responsibilities further limited implementation. Collectively, these findings suggest that preventive behavior is influenced not by a single HBM construct but by the interaction of multiple perceptions. Even when awareness, perceived severity, and perceived benefits are high, preventive actions may remain suboptimal if individuals perceive themselves to be at low personal risk, encounter substantial barriers, or lack confidence in their ability to adopt and sustain recommended behaviors.

Most physicians did not agree that cancer screening is only necessary in the presence of symptoms, indicating appropriate understanding of preventive screening principles. Participants reported strong cues to action, particularly in response to health campaign reminders and institutional wellness initiatives. Nevertheless, participation in cancer awareness campaigns was limited, especially among non-oncology physicians indicating that passive exposure to educational interventions does not always translate into sustained involvement in preventive activities unless accompanied by supportive systems and incentives^42^.

### Implications for practice

-The findings indicate that strategies to improve cancer prevention among physicians should extend beyond educational interventions.

-Institutional policies that support physician wellness, facilitate access to screening services, and incorporate reminder systems may help reduce practical barriers as follows:

- **Institutional Reforms**: To mitigate severe time constraints and the workplace guilt of leaving busy shifts, hospitals should implement mandatory, protected “wellness hours” and “health days” specifically designated for routine screenings. Additionally, establishing confidential, fast tracked screening pathways or partnering - with external networks to alleviate anxieties regarding privacy and peer-to-peer “role reversal.”
- **Policy and Structural Interventions**: National health policies should improving access to HPV vaccination for healthcare workers by providing fully subsidized, workplace-integrated adult vaccination programs.
- **Educational Integration**: Medical boards and institutional leadership should incentivize personal health by integrating self-care metrics and preventive guidelines into Continuing Medical Education (CME) credits, using peer-led advocacy (particularly from oncology specialties) to drive cultural change across non-oncology departments.

### Strengths and limitations

The study’s strengths include its comprehensive assessment of multiple dimensions of cancer prevention and the inclusion of physicians from diverse specialties. A notable strength and novel aspect of the present study is its assessment of cancer prevention practices not only among physicians themselves but also within their families. While previous studies have predominantly evaluated physicians’ knowledge, attitudes, and personal screening behaviors, few have explored the extent to which physicians promote cancer prevention among their partners and family members. Given that physicians often serve as trusted sources of health information within their social networks, their influence may extend beyond patient care to shaping preventive behaviors among relatives. The findings therefore provide a broader understanding of how professional knowledge is translated into preventive actions within both personal and family contexts.

While this study offers valuable insights into the health behaviors of medical professionals, several limitations must be acknowledged. The cross-sectional design limits causal inference, and the reliance on self-reported data may introduce reporting bias. The reliance on self-reported data introduces the potential for social desirability bias, as physicians may over report positive health behaviors (such as screening or healthy dietary intake) to align with professional expectations. Additionally, retrospective reporting of remote behaviors, such as older vaccination records or distant screening dates, may introduce recall bias.

Selection and Geographic Bias: The study sample predominantly features physicians from urban and university-affiliated medical centers. While this cohort provides excellent insight into highly specialized environments, university-affiliated physicians generally have more uniform access to clinical guidelines, training, and resources. Consequently, these findings may not fully generalize to physicians practicing in rural settings, private solo practices, or community clinics where structural barriers and resource access differ substantially.

The statistical analysis primarily focused on descriptive and bivariate methods to explore physicians’ cancer prevention practices and their associations with demographic and professional characteristics. Although these analyses provide valuable exploratory insights, the absence of multivariable regression models limits the ability to identify independent predictors and control for potential confounding factors. Future studies with larger sample sizes are warranted to incorporate multivariable analyses and further investigate determinants of cancer prevention behaviors among physicians.

## Conclusion

Despite high professional awareness, physicians demonstrated limited engagement in personal cancer screening and preventive practices. Oncology-related practice enhanced preventive advocacy toward family members but did not translate into improved personal behaviors. These findings highlight a persistent knowledge–practice gap and the need for institutional strategies that facilitate physician wellness, screening access, and preventive engagement.

Addressing this gap requires a shift in intervention strategies from passive education to structural workplace reforms, such as introducing mandatory, confidential physician wellness days and on-site, subsidized adult immunization initiatives. Concurrently, future studies should employ qualitative approaches to explore the psychological barriers to physician self-care including invincibility bias and privacy concerns while using longitudinal designs to assess how institutional health programs impact physician screening adherence and clinical advocacy.

## Supplementary Information

Below is the link to the electronic supplementary material.

Supplementary Material 1

## Data Availability

Data are available upon reasonable request from the corresponding author.

## Abbreviations

HBM: Health Belief Model
HCPs: Healthcare providers
HPV: Human papilloma virus
PSA: Prostatic specific antigen
CT: Computed tomography
IRB: Institutional Review Board
