# Supplementary material for: Personal and family application of cancer prevention practices among physicians
Source: Sci Rep. 2026 Aug 18;16:25891. doi: 10.1038/s41598-026-66073-4 (PMC13486735; doi:10.1038/s41598-026-66073-4)
Supplement: Supplementary file 1 [file 41598_2026_66073_MOESM1_ESM.docx]

**Annex I (Supplementary File 1): Questionnaire Health Belief Model Constructs of Personal and Family Application of Cancer Prevention Practices Among Physicians in Egypt**

|  | **No. Items** | **Cronbach’s alpha** |
| --- | --- | --- |
| **Perceived Susceptibility** | 2 | 0.60 |
| I believe I am at risk of developing cancer in my lifetime. |  |  |
| Cancer could affect anyone regardless of profession. |  |  |
| **Perceived Severity** | 2 | 0.82 |
| Cancer has serious consequences for my life and family. |  |  |
| If I were diagnosed with cancer, my career would be negatively affected. |  |  |
| **Perceived Benefits** | 2 | 0.88 |
| Following cancer prevention guidelines can significantly reduce my risk. |  |  |
| Screening helps detect cancer early and improves outcomes. |  |  |
| **Perceived Barriers** | 5 | 0.60 |
| I don’t have time to engage in screening or healthy practices. |  |  |
| I am concerned about the cost of cancer screening. |  |  |
| I avoid screening because I don’t want to know bad news. |  |  |
| **Self-Efficacy** | 2 | 0.80 |
| I feel confident in my ability to follow cancer prevention guidelines. |  |  |
| I can influence my family’s health-related behaviors positively. |  |  |
| **Cues to Action** | 2 | 0.82 |
| I am more likely to act if I receive reminders from health campaigns. |  |  |
| Seeing patients with cancer makes me more aware of prevention needs. |  |  |
